# Relative Contributions of Gastrointestinal‐Specific Hypervigilance and Anxiety in Explaining Eating Disorder Symptoms

**DOI:** 10.1002/jclp.70134

**Published:** 2026-03-27

**Authors:** Gabriella Pucci, Naomi G. Hill, Zoe Schneider, Tiffany A. Brown, Helen Burton‐Murray, Livia Guadagnoli, K. Jean Forney

**Affiliations:** ^1^ Department of Psychology Ohio University Athens Ohio USA; ^2^ Carnegie Mellon University Pittsburgh Pennsylvania USA; ^3^ Department of Psychological Sciences Auburn University Auburn Alabama USA; ^4^ Division of Gastroenterology, Center for Neurointestinal Health, Massachusetts General Hospital Boston Massachusetts USA; ^5^ Harvard Medical School Boston Massachusetts USA; ^6^ Department of Gastroenterology and Hepatology Northwestern Feinberg School of Medicine Chicago Illinois USA

**Keywords:** anxiety, feeding and eating disorders, gastrointestinal symptoms, hypervigilance, purging, restriction

## Abstract

**Objective:**

Gastrointestinal (GI) disturbances and eating disorder symptoms commonly co‐occur. Hypervigilance and anxiety about GI symptoms may motivate eating disorder behaviors to manage and/or avoid GI symptoms. We hypothesized that GI‐specific anxiety would be more strongly associated with dietary restriction and purging than GI‐specific hypervigilance. Further, we hypothesized that distinguishing between GI‐specific anxiety and hypervigilance would have incremental validity over using an existing measure of GI‐anxiety and related constructs initially developed for patients with irritable bowel syndrome, the Visceral Sensitivity Index.

**Methods:**

Three hundred and eighty‐two undergraduate students with elevated eating pathology (83.5% female, 87.4% White) completed questionnaires assessing GI‐specific hypervigilance and anxiety, the Visceral Sensitivity Index, and eating disorder symptoms. Participants were recruited with elevated shape/weight‐oriented eating pathology (*N* = 309) and elevated avoidant/restrictive food intake disorder‐related eating pathology (*N* = 73). Analyses were pre‐registered.

**Results:**

Regarding dietary restriction, the relative weight of GI‐specific anxiety did not differ from that of GI‐specific hypervigilance. Further, neither variable provided incremental validity over the Visceral Sensitivity Index in understanding restriction. Regarding purging, GI‐specific anxiety was not more strongly associated with purging than GI‐specific hypervigilance. However, GI‐specific anxiety, but not GI‐specific hypervigilance, demonstrated incremental validity over the Visceral Sensitivity Index in explaining purging.

**Conclusion:**

Distinguishing between GI‐specific hypervigilance and anxiety adds incremental validity in understanding purging but not restriction. Future research should examine whether addressing anxious cognitions about GI symptoms improves purging outcomes.

## 1 Introduction

Gastrointestinal (GI) disturbances and eating disorder symptoms are highly comorbid in both shape and weight‐oriented eating disorders (e.g., bulimia nervosa) and avoidant/restrictive food intake disorder (ARFID) (Mikhael‐Moussa et al. 2025; Peters et al. 2022; Riedlinger et al. 2020). Both GI disturbances and eating disorder symptoms are theorized to bidirectionally influence one another (Frank et al. 2021; Wildes et al. 2021). GI‐specific hypervigilance (e.g., continuous monitoring of gut sensations) and anxiety (e.g., catastrophic thinking or rumination) may underlie the proposed link between GI disturbances and eating disorder behaviors (Wildes et al. 2021), such that monitoring of and worry about GI symptoms contribute to engagement in eating disorder behaviors. Prior work using the Visceral Sensitivity Index (Labus et al. 2004), a measure initially developed to assess lower‐GI worry, fear, vigilance, sensitivity, and avoidance, has suggested that GI‐specific anxiety is positively associated with eating disorder symptoms in eating disorder and chronic constipation populations (Brown et al. 2021; Murray et al. 2020) as well as college populations and MTurk workers (Poovey et al. 2024; Zickgraf and Ellis 2018). Early change in the Visceral Sensitivity Index also predicts better eating disorder treatment outcomes at discharge from a partial hospitalization program and at 6‐month follow‐up (Velkoff et al. 2024). Problematically, the Visceral Sensitivity Index contains item content that mostly pertains to lower‐GI problems (e.g., references the belly and bowel movements), while patients with eating disorders experience both lower‐GI problems (e.g., constipation) and upper‐GI problems (e.g., heartburn, reflux; Riedlinger et al. 2020). Further, the Visceral Sensitivity Index assesses a conglomerate construct and does not differentiate between fear‐related constructs such as anxiety and hypervigilance, potentially limiting precision and interpretability (Strauss and Smith 2009).

The Psychobiological Model of Disorders of Gut–Brain Interaction (Guadagnoli et al. 2025), which builds upon the Fear‐Avoidance Model of Pain (Vlaeyen et al. 2016), distinguishes between attentional processes (e.g., interoceptive threat monitoring; hypervigilance), cognitive and affective features (e.g., emotional distress, catastrophizing, anxiety), and behavior (e.g., avoidance) as contributors to bothersome gastrointestinal sensations. For individuals with eating disorders, behaviors like dietary restriction (i.e., avoiding certain foods, avoiding fullness) and purging (i.e., removing uncomfortable stimuli) may serve an avoidance function to minimize gastrointestinal sensations. According to the model, emotional distress (e.g., anxiety, catastrophizing, worry) is directly linked to interference, or impairment from avoidance behaviors, whereas interoceptive threat monitoring (e.g., hypervigilance) is not. Instead, hypervigilance is implicated in symptom perception severity.

Distinguishing between hypervigilance and anxiety has promise for clinical utility in GI disorders. Esophageal hypervigilance, but not anxiety, was uniquely associated with esophageal symptom severity in gastroenterology outpatients with reflux symptoms (e.g., heartburn, regurgitation; Guadagnoli et al. 2021), while esophageal anxiety, but not hypervigilance, was uniquely associated with poorer quality of life and increased symptoms in patients referred for surgical evaluation of gastroesophageal reflux disease (Hill et al. 2021). Both hypervigilance and anxiety are independently associated with dysphagia severity and quality of life in eosinophilic esophagitis, with relative effect sizes suggesting a greater importance for anxiety (Taft et al. 2021). Identifying an individual's most relevant maintaining symptoms (e.g., anxiety or hypervigilance), and then tailoring psychological interventions to focus on the individual's most relevant maintaining symptoms (e.g., cognitive restructuring for catastrophizing, muscle relaxation for hypervigilance; Guadagnoli et al. 2025; Keefer and Mandal 2015) may improve treatment outcomes for GI symptoms in eating disorders.

We previously evaluated the psychometric properties of a modified version of the Esophageal Hypervigilance and Anxiety Scale (EHAS; Taft et al. 2018) to assess GI‐specific hypervigilance and anxiety across the entire gut (Forney et al. 2024), building upon the limitations of the Visceral Sensitivity Index (Brown et al. 2021; Murray et al. 2020; Velkoff et al. 2024). Post hoc analyses found that GI‐specific anxiety was more strongly related to GI and general somatic symptoms than GI‐specific hypervigilance in our sample of individuals with elevated eating pathology. However, associations with eating pathology were not examined. Our findings suggest GI‐specific anxiety might be most relevant for individuals with elevated eating pathology, consistent with findings from GI disorders (Hill et al. 2021; Taft et al. 2021) and the Psychobiological Model of Disorders of Gut–Brain Interaction.

The current study builds upon prior work to test the hypothesis that GI‐specific anxiety will be more strongly related to eating pathology than GI‐specific hypervigilance using relative weights analysis. We focus specifically on understanding dietary restriction and purging, as these eating disorder symptoms are most proximally related to meals and have previously been associated with GI symptoms (Forney, Horvath, et al. 2023) and the Visceral Sensitivity Index (Brown et al. 2021; Poovey et al. 2024). We also tested the hypothesis that the EHAS, modified to assess both lower and upper GI concerns, would have incremental validity in understanding dietary restriction and purging over the Visceral Sensitivity Index. This is the first study to evaluate how the modified EHAS is related to eating pathology and to test its incremental validity relative to the Visceral Sensitivity Index. We included individuals with both shape/weight‐oriented eating pathology and individuals with ARFID‐related eating pathology, given that gastrointestinal symptoms are common across these diagnostic groups (Mikhael‐Moussa et al. 2025; Riedlinger et al. 2020).

## 2 Materials and Methods

### 2.1 Participants

The following secondary data analysis was pre‐registered (https://doi.org/10.17605/OSF.IO/EWS4C). Data come from a study that sought to evaluate the psychometric properties of a modified version of the EHAS in individuals with elevated eating pathology (Forney et al. 2024). There were no eligibility requirements related to gastrointestinal symptoms. All participants (*N* = 382 university students; *M*_age_ = 19.96, SD = 3.99 years) from the parent study were included. Participants' racial identities were White (87.4%, *N* = 334), Black or African American (4.2%, *N* = 16), Asian or Asian American (2.4%, *N* = 9), multiracial (2.4%, *N* = 9), Native Hawaiian or Pacific Islander (0.5%, *N* = 2), and three participants (0.8%) preferred to self‐describe their racial identity using an open‐ended response option. Ten participants (2.6%) identified as Hispanic or Latino. Eight participants (2.1%) were missing data for racial/ethnic identity. Participants' gender identities were female (83.5%, *N* = 319), male (14.7%, *N* = 56), and gender expansive (i.e., transgender, genderqueer, or self‐describing; 1.8%, *N* = 7). Participants' sexual orientations were heterosexual (78.5%, *N* = 300), bisexual (14.7%, *N* = 56), gay or lesbian (2.9%, *N* = 11), and 15 participants (3.9%) preferred to self‐describe.

### 2.2 Procedure

University students were recruited through psychology department research pools at two public universities in the United States. Data collection occurred from March 2021 through April 2022. Participants provided informed consent, completed online surveys at two time points, and were compensated with partial course credit. All procedures were approved through the university's institutional review boards.

At the first university, participants were invited to participate if they screened positive for elevated eating pathology, and eligibility was confirmed at Time 1 (see Measures – Eligibility Criteria). There were no screening criteria at the second university; however, only participants who met the screening criteria were included in analyses. Across both sites, participants were excluded from analyses if they (1) spent less than 2 s per survey item or (2) failed more than half of the insufficient effort responding items (Curran 2016; Huang et al. 2015). Participants repeated some questionnaires 2 weeks later. Only data from Time 1 were used in the present study.

### 2.3 Measures

#### 2.3.1 Eligibility Measures

Participants were classified as having elevated eating pathology if they met the following criteria for either (1) elevated shape/weight‐oriented eating pathology or (2) elevated ARFID‐related eating pathology.

Participants were classified as having elevated shape/weight‐oriented eating pathology if they scored at or above 3.88 on the Eating Disorder Examination Questionnaire‐8 (EDE‐Q8; Kliem et al. 2016; Machado et al. 2020) or if they endorsed engaging in eating disorder behaviors (e.g., binge eating, self‐induced vomiting, laxative misuse, or compulsive exercise) four or more times in the past month using the behavioral items from the EDE‐Q (Fairburn and Beglin 1994).

Participants were classified as having elevated ARFID‐related eating pathology (i.e., restriction of food intake due to sensory sensitivity, fear of aversive consequences, and/or lack of interest) if they scored above any of the following cut‐off scores on the Nine Item Avoidant/Restrictive Food Intake Disorder Screen (Zickgraf and Ellis 2018) *and* scored less than 2.3 on the EDE‐Q8: (1) ≥ 10 on Picky Eating/Sensory, (2) ≥ 9 on Lack of Interest/Low Appetite, and (3) ≥ 10 on Fear of Aversive Consequences (Burton Murray et al. 2021).

#### 2.3.2 Primary Measures

**Modified EHAS** (Forney et al. 2024). The EHAS (Taft et al. 2018) was modified to assess hypervigilance and anxiety across the entire gastrointestinal tract (e.g., both lower and upper GI symptoms) rather than only esophageal symptoms (Forney et al. 2024). In the validation paper using the same dataset, a two‐factor model (i.e., GI‐specific hypervigilance and GI‐specific anxiety) fit better than a one‐factor model, supporting distinguishing between the constructs. The modified EHAS generally exhibited good convergent validity and excellent discriminant validity. Test‐retest reliability of the modified EHAS across 2 weeks in the present sample was good (ICC Hypervigilance = 0.64; Anxiety = 0.77). Cronbach's alpha was good across the full sample and weight/shape‐oriented eating pathology sample (Hypervigilance = 0.86–0.87 and Anxiety = 0.92).

**Eating Pathology Symptoms Inventory** (EPSI; Forbush et al. 2013). The Restricting and Purging subscales were used in the present study. These subscales exhibit moderate to strong associations with existing measures of eating pathology, supporting convergent validity and low correlations with measures of depression and anxiety symptoms, supporting discriminant validity (Forbush et al. 2013). The Restricting subscale does not reference shape or weight motivations for restrictive eating behaviors, making it appropriate for assessing restricting in ARFID‐like presentations. Indeed, patients with anorexia nervosa and ARFID have comparable Restricting subscale scores (Coniglio et al. 2018). Test‐retest reliability in a sample of college students across a 2‐ to 4‐week period was good (ICC Restricting = 0.75; Purging = 0.73; Forbush et al. 2013). Cronbach's alpha was good for Restricting (0.87) and Purging (0.83). The EPSI instructions were modified to be administered online with the copyright holder's permission.

**Visceral Sensitivity Index** (Labus et al. 2004). The Visceral Sensitivity Index is a 15‐item questionnaire that assesses worry, fear, catastrophizing, vigilance, sensitivity, and avoidance about sensations in the lower GI tract (e.g., “I am constantly aware of the feelings I have in my belly”). In the parent study (Forney et al. 2024), the 13‐item Visceral Sensitivity Index exhibited large associations with the hypervigilance and anxiety subscales of the modified EHAS (*r* = 0.64 and *r* = 0.81, respectively). The 15‐item version demonstrates large associations with irritable bowel syndrome symptom severity in an irritable bowel syndrome study population (Labus et al. 2004), supporting convergent validity. Discriminant validity is supported by small associations with desire to gain more muscle within an eating disorder sample comprising both weight/shape‐oriented eating disorders and ARFID (Brown et al. 2021) and muscle‐building behaviors in a college population (Poovey et al. 2024). The original, 15‐item Visceral Sensitivity Index scoring was used; Cronbach's alpha was excellent across subsamples (Cronbach's alpha = 0.92).

**Insufficient Effort Responding** (Huang et al. 2015). This eight‐item measure was used to assess sufficient effort responding. Each item was embedded within the larger survey. Participants who endorsed more than half of the improbable items (e.g., “I can run 2 miles in 2 minutes”) were excluded from the dataset (Forney et al. 2024).

### 2.4 Data Analytics Plan

Our data analytic plan was pre‐registered after publication of the parent study (https://doi.org/10.17605/OSF.IO/EWS4C). De‐identified data are publicly available (https://osf.io/gh624/files/osfstorage). Relative weights analysis (Tonidandel and LeBreton 2015) tested both hypotheses using a Shiny web app (https://rwa-web.shinyapps.io/multipleregression/) with the following settings: (1) pairwise deletion, (2) 10,000 bootstrap replications, and (3) alpha = 0.05. Missing data were minimal (*n* = 2 for EPSI; *n* = 1 for Visceral Sensitivity Index) and handled using pairwise deletion. In relative weights analysis, predictor variables are transformed to be orthogonal with one another, allowing the total variance to be decomposed into unique contributions for each variable. To examine the unique contributions of GI‐specific hypervigilance and anxiety on restriction or purging, two models were run, one with Restricting as the dependent variable and one with Purging as the dependent variable. We evaluated whether the relative weights were statistically significant from zero and if the relative weights were statistically different from one another. The same two models were run with Visceral Sensitivity Index scores also included in the model to examine whether distinguishing between GI‐specific hypervigilance and anxiety has incremental validity in predicting restriction or purging. Data from all participants were used in analyses examining restriction. Only data from those with elevated shape/weight‐oriented eating pathology with complete data (*N* = 309) were evaluated for analyses examining purging, as purging behaviors to influence shape and weight are not part of the ARFID clinical presentation.

During the review process, we decided to deviate from our planned registration after reviewer feedback. A large minority (42.1%) of participants in the elevated shape/weight eating pathology sample had scores of 0 on the Purging subscale, and 18% (*n* = 55) endorsed engaging in purging behaviors over the prior 28 days on the EDE‐Q behavioral items. Given evidence of skewness (1.966) and kurtosis (4.474) and some evidence for non‐normality of residuals in analyses using the untransformed Purging subscale, we report analyses with a square‐root transformation of the purging scores. The untransformed scores, which were pre‐registered, are reported in Table S1. Interpretation of the results is the same.

## 3 Results

Table 1 presents descriptive information and bivariate correlations amongst study variables. Both restricting and purging demonstrated small correlations with GI‐specific anxiety and hypervigilance.

**Table 1:** Descriptive information and bivariate correlations between study variables in a sample of undergraduate students with elevated eating pathology.

Variable	1	2	3	*M*	SD	Observed range	*N*
1. Modified EHAS‐ Hypervigilance Subscale^a^	—	—	—	11.96	6.15	0–24	382
2. Modified EHAS‐ Anxiety Subscale^a^	0.719**	—	—	12.80	9.45	0–36	382
3. Visceral Sensitivity Index^a^	0.618**	0.792**	—	28.10	16.37	0–72	381
4. Eating Pathology Symptoms Inventory Restricting^a^	0.125*	0.187**	0.239**	8.60	5.67	0–24	380
5. Eating Pathology Symptoms Inventory Purging (untransformed)^b^	0.125*	0.199**	0.222**	3.07	4.32	0–24	309
6. Eating Pathology Symptoms Inventory Purging (square root transformation)^b^	0.121*	0.197**	0.238**	1.23	1.25	0–4.90	309

Abbreviation: EHAS, Esophageal Hypervigilance and Anxiety Scale.

^a^ Full sample.

^b^ Shape/weight sample only.

* *p* < 0.05;

** *p* < 0.001.

### 3.1 Dietary Restriction

In the full sample, GI‐specific anxiety and GI‐specific hypervigilance jointly explained 3.5% of the variance in restriction (Table 2). GI‐specific anxiety, but not GI‐specific hypervigilance, explained significant variance in Restricting scores. The relative weight of GI‐specific anxiety was three times larger than, but not significantly different from, the relative weight of GI‐specific hypervigilance (95% CI [−0.001, 0.052]).

**Table 2:** Relative weights analysis examining the contributions of gastrointestinal‐specific hypervigilance and anxiety to restriction in a sample of undergraduate students (*n* = 380) with elevated eating pathology.

	Raw relative weight estimate [95% CI]	95% CI for the significance of the relative weight
Hypervigilance	0.008 [0.001, 0.022]	−0.006, 0.029
Anxiety	0.027 [0.006, 0.062]	0.003, 0.066
*R* ^2^	0.035	
Hypervigilance	0.006 [0.001, 0.011]	−0.015, 0.014
Anxiety	0.016 [0.004, 0.034]	−0.005, 0.037
Visceral Sensitivity Index	0.037 [0.010, 0.078]	0.007, 0.080
*R* ^2^	0.058	

*Note:* The raw relative weight indicates the proportion of variance in restricting that is explained by each predictor.

When the Visceral Sensitivity Index was added to the model, GI‐specific hypervigilance, GI‐specific anxiety, and the Visceral Sensitivity Index jointly explained 5.8% of the variance in restriction. Only the Visceral Sensitivity Index contributed statistically significant variance to restriction. The relative weights of GI‐specific anxiety and GI‐specific hypervigilance were not statistically different from one another (95% CI [−0.031, 0.001]).

### 3.2 Purging

In participants with elevated shape/weight‐related eating pathology, GI‐specific anxiety and GI‐specific hypervigilance jointly explained 4.0% of the variance in purging. GI‐specific anxiety, but not GI‐specific hypervigilance, explained significant variance in purging (Table 3). The relative weight of GI‐specific anxiety was four times larger, though not significantly different from, the relative weight of GI‐specific hypervigilance (95% CI [0.000, 0.067]).

**Table 3:** Relative weights analysis examining the contributions of gastrointestinal‐specific hypervigilance and anxiety to purging (square root transformed) in a sample of undergraduate students (*n* = 309) with elevated weight and shape‐oriented eating pathology.

	Raw relative weight estimate [95% CI]	95% CI for the significance of the relative weight
*Purging (square root transformed)*		
Hypervigilance	0.008 [0.001, 0.021]	−0.006, 0.029
Anxiety	0.032 [0.006, 0.080]	0.004, 0.084
*R* ^2^	0.040	
Hypervigilance	0.006 [0.001, 0.010]	−0.010, 0.018
Anxiety	0.018 [0.005, 0.047]	0.001, 0.052
Visceral Sensitivity Index	0.036 [0.009, 0.078]	0.005, 0.083
*R* ^2^	0.060	

*Note:* The raw relative weight indicates the proportion of variance in purging that is explained by each predictor.

When the Visceral Sensitivity Index was added to the model, GI‐specific hypervigilance, GI‐specific anxiety, and the Visceral Sensitivity Index jointly explained 6.0% of the variance in purging. Both GI‐specific anxiety and the Visceral Sensitivity Index contributed significant variance to purging. The relative weights of GI‐specific anxiety and GI‐specific hypervigilance were not significantly different from one another (95% CI [0.00, 0.043]).

### 3.3 Post Hoc Analyses

Because the diverging results between restriction and purging could reflect differences in samples, we re‐ran the dietary restriction analyses in the elevated shape/weight eating pathology subsample. Neither GI‐specific anxiety nor hypervigilance contributed significant variance to restriction, and the relative weights were not statistically different from one another (95% CI [−0.002, 0.054]), including when the Visceral Sensitivity Index was included in the model (95% CI [−0.002, 0.032]). We also re‐ran the dietary restriction analyses in the ARFID‐only eating pathology subsample. Neither GI‐specific anxiety nor hypervigilance contributed significant variance to restriction, and the relative weights were not statistically different from one another (95% CI [−0.029, 0.119]), including when the Visceral Sensitivity Index was included in the model (95% CI [−0.042, 0.062]).

The modified EHAS GI‐specific anxiety subscale shares four items with the Visceral Sensitivity Index, although the scales vary in whether items probe about the “gut” (the modified EHAS) or “belly” (Visceral Sensitivity Index). To conduct an even stronger test of the incremental validity of the modified EHAS, we removed the shared items from the modified EHAS and re‐ran analyses (see Table S2). Similar to our primary analyses, only the Visceral Sensitivity Index explained significant variance in dietary restriction. No variables explained significant variance in the square root transformed purging variable.

Our pre‐registered analyses focused on dietary restriction and purging, as these behaviors are most proximally related to meals and have previously been associated with GI symptoms (Forney, Horvath, et al. 2023) and the Visceral Sensitivity Index (Brown et al. 2021; Poovey et al. 2024). We ran exploratory analyses examining binge eating and cognitive restraint (see Table S3). GI‐specific anxiety, but not GI‐hypervigilance, contributed significant variance in binge eating. When the Visceral Sensitivity Index was added to the model, both GI‐specific anxiety and the Visceral Sensitivity Index contributed significant variance in binge eating. No variables contributed significant variance to cognitive restraint.

The Visceral Sensitivity Index had the largest relative weights in understanding both restricting and purging. Given that the Visceral Sensitivity Index was developed and validated in a sample of patients with irritable bowel syndrome (Labus et al. 2004), this could reflect that lower GI symptoms are more strongly associated with eating disorder symptoms than upper GI symptoms. We ran additional analyses exploring associations between specific upper and lower GI symptoms and restricting and purging using the Patient Assessment of Gastrointestinal Symptom Severity Index (Rentz et al. 2004) and the Patient Assessment of Constipation Symptom Severity Index (Frank et al. 1999; see Table S4). There were no instances in which lower GI symptoms were more strongly correlated with restricting than upper GI symptoms. There was only one instance in which lower GI symptoms were more significantly correlated with purging than upper GI symptoms: constipation‐related abdominal symptoms were more strongly correlated with purging than postprandial fullness/early satiety. In contrast, some correlations with restricting and purging were statistically larger for upper GI symptoms compared to lower GI symptoms.

## 4 Discussion

The current study tested the hypothesis that GI‐specific anxiety would be a stronger correlate of dietary restriction and purging than GI‐specific hypervigilance among individuals with elevated eating pathology. We also examined whether distinguishing between GI‐specific hypervigilance and anxiety using a measure adapted for the entire GI tract would have incremental validity over the Visceral Sensitivity Index in understanding restriction and purging. Our hypotheses were partially supported. GI‐specific anxiety, but not hypervigilance, was a significant correlate of restriction; however, these relative weights were not statistically different from one another. Neither GI‐specific anxiety nor GI‐specific hypervigilance had incremental validity above the Visceral Sensitivity Index in explaining restriction. Similarly, GI‐specific anxiety, but not GI‐specific hypervigilance, was a correlate of purging; these relative weights were not statistically different from one another. When the Visceral Sensitivity Index was added to the model, GI‐specific anxiety continued to explain a significant amount of variance in purging, supporting the incremental validity of GI‐specific anxiety in predicting purging, but not restriction.

GI‐specific anxiety, but not GI‐specific hypervigilance, was a unique correlate of purging and had incremental validity over the Visceral Sensitivity Index in explaining purging, albeit the relative effect sizes were small. Given that the Visceral Sensitivity Index and modified EHAS share four items, the small incremental validity represents a somewhat robust effect. When removing the shared items from the GI‐specific anxiety measure, neither the Visceral Sensitivity Index nor GI‐specific anxiety was significantly associated with purging, although the confidence intervals approached excluding 0 for both variables. Pending replication, targeting GI‐specific anxiety may improve treatment outcomes in eating disorders marked by purging behaviors. Multiple studies have demonstrated that the severity of GI symptoms typically decreases with eating disorder treatment (Burton Murray et al. 2023; Forney, Burton Murray, et al. 2023; Salvioli et al. 2013; West et al. 2021), with greater decreases in postprandial fullness observed for those higher in trait anxiety (Forney, Burton Murray, et al. 2023). Augmenting eating disorder treatments with psychoeducation about expected reductions in GI symptoms or incorporating exposures targeting gastric sensations (Boswell et al. 2019) should be evaluated as tools to challenge catastrophic, anxious cognitions about GI symptoms in order to ultimately reduce purging.

GI‐specific anxiety and GI‐specific hypervigilance did not provide incremental validity in understanding restriction. Instead, only the Visceral Sensitivity Index contributed significant variance. Post hoc analyses indicate the pattern of results was the same across diagnostic subgroups, suggesting the discrepant results for restriction do not reflect sample composition. While purging typically occurs in response to food intake and may reduce feared post‐meal GI symptoms, restriction may be used to prevent feared GI sensations from developing in the first place. The larger relative weight for the Visceral Sensitivity Index may reflect that the Visceral Sensitivity Index includes avoidance‐related content, whereas avoidance is not represented in the measure of GI‐specific anxiety and hypervigilance. If dietary restriction represents an avoidance behavior intended to minimize GI symptoms, interventions framed to directly target avoidance, such as behavioral experiments and exposures in cognitive behavioral therapy, may be valuable for reducing restriction (Boswell et al. 2019; Schaumberg et al. 2021).

We also explored the relationships between GI‐specific anxiety and hypervigilance with binge eating and cognitive restraint. Similar to our purging results, GI‐specific anxiety, but not hypervigilance, was related to binge eating. Similar to our restriction results, neither GI‐specific anxiety nor hypervigilance was related to cognitive restraint. These patterns may reflect high comorbidity between binge eating and purging. Alternatively, the relationship between GI‐specific anxiety and binge eating may reflect that anxiety‐prone individuals may be more likely to interpret overeating as “loss of control” eating if the overeating leads to uncomfortable GI sensations.

Overall, the Visceral Sensitivity Index was the most robust correlate of eating pathology. While the Visceral Sensitivity Index was developed in a sample with lower‐GI problems (Labus et al. 2004), our pattern of results does not suggest that lower GI‐problems are more salient to eating disorder behaviors. Our post hoc analyses indicated that, in some instances, upper GI‐problems were more strongly linked to eating disorder behaviors than lower GI‐problems. Instead, the robustness of the Visceral Sensitivity Index may reflect that other constructs tapped by this conglomerate measure, such as sensitivity, are more relevant to eating disorder behaviors than anxiety and hypervigilance. Alternatively, the items on the Visceral Sensitivity Index intended to tap into GI‐related concerns (“I get anxious when I go to a new restaurant”) are tapping into eating disorder‐related fears, artificially inflating associations. Strauss and Smith (2009) argue that homogeneous measures, such as the subscales of the modified EHAS, provide better precision and therefore may be more useful to researchers and clinicians alike than conglomerate measures. More work is needed to understand how to best assess GI‐related constructs within eating disorders.

The current study benefited from the use of pre‐registration, a sample with elevated eating pathology, and a separate examination of GI‐specific hypervigilance and anxiety. Our shape/weight‐oriented eating pathology subsample had a relatively high representation of purging symptoms (e.g., 18% endorsed purging behaviors in the past 28 days) and a significant minority (27%) self‐reported being diagnosed with a GI condition. We also observed meaningful variability in GI symptom measures (Forney et al. 2024), limiting concerns that the findings are driven by low variability. Limitations of this study include its cross‐sectional nature, which restricts insight into the directionality of these relations, the lack of medical evaluation to detect GI disorders, and the constraints of self‐report to assess eating pathology. The present sample was primarily female and non‐Hispanic White; it is unknown if results would generalize to a more diverse sample.

In summary, the current study suggests there may be utility in distinguishing between GI‐specific anxiety and hypervigilance when examining purging. Given the relatively small effects, it may be the case that associations between GI symptoms and eating disorder behaviors reflect a third variable. One possible third variable is comorbid anxiety, which was not measured in this study. Indeed, internalizing disorders, GI symptoms, and eating disorders share significant genetic etiology (Mikhail et al. 2024). Longitudinal designs and multi‐modal assessments of anxiety and hypervigilance are needed to understand how concerns around GI symptoms may be implicated in the maintenance of eating disorders or whether these are both correlates of underlying anxiety. Pending positive results, future intervention research may wish to investigate whether GI‐specific anxiety has utility in reducing purging.

## Author Contributions

K. Jean Forney, Gabriella Pucci, Tiffany A. Brown, Helen Burton‐Murray, and Livia Guadagnoli conceptualized the project. K. Jean Forney, Tiffany A. Brown, and Gabriella Pucci supervised data collection. Naomi G. Hill conducted statistical analyses. Gabriella Pucci, Naomi G. Hill, Zoe Schneider, and K. Jean Forney drafted the manuscript. All authors reviewed and edited the manuscript.

## Ethics Statement

Data collection was approved by the institutional review boards at Ohio University (20‐F‐32) and Auburn University (21‐413).

## Conflicts of Interest

H.B.‐M. receives royalties from Oxford University Press for her book *Cognitive Behavioral Therapy for Rumination Syndrome* and from Cambridge University Press for her forthcoming book on functional bowel disorders. The other authors declare no conflicts of interest.

## Supporting information


**Table 1:** Relative weights analysis examining the contributions of gastrointestinal‐specific hypervigilance and anxiety to purging in a sample of undergraduate students (n=309) with elevated weight and shape‐oriented eating pathology. **Table 2:** Relative weights analysis examining the incremental validity of the unique modified EHAS Anxiety subscale items over and above the modified EHAS Hypervigilance subscale and the Visceral Sensitivity Index in explaining restricting (full sample, n=380) and purging (shape/weight‐oriented eating pathology subsample, n=309). **Table 3:** Exploratory relative weights analysis examining the contributions of gastrointestinal‐specific hypervigilance and anxiety to binge eating and cognitive restraint in a sample of undergraduate students (n=309) with elevated weight and shape‐oriented eating pathology. **Table 4:** Correlations between gastrointestinal symptoms and eating disorder symptoms in a sample of undergraduate students with elevated eating pathology.

## Data Availability

Data are publicly available at https://osf.io/gh624/files/osfstorage.
