# Supplementary material for: Relative Contributions of Gastrointestinal‐Specific Hypervigilance and Anxiety in Explaining Eating Disorder Symptoms
Source: J Clin Psychol. 2026 Mar 27;82(7):1078–85. doi: 10.1002/jclp.70134 (PMC13104586; doi:10.1002/jclp.70134)
Supplement: Supplementary file 1 — Table 1: Relative weights analysis examining the contributions of gastrointestinal‐specific hypervigilance and anxiety to purging in a sample of undergraduate students (n=309) with elevated weight and shape‐oriented eating pathology. Table 2: Relative weights analysis examining the incremental validity of the unique modified EHAS Anxiety subscale items over and above the modified EHAS Hypervigilance subscale and the Visceral Sensitivity Index in explaining restricting (full sample, n=380) and purging (shape/weight‐oriented eating pathology subsample, n=309). Table 3: Exploratory relative weights analysis examining the contributions of gastrointestinal‐specific hypervigilance and anxiety to binge eating and cognitive restraint in a sample of undergraduate students (n=309) with elevated weight and shape‐oriented eating pathology. Table 4: Correlations between gastrointestinal symptoms and eating disorder symptoms in a sample of undergraduate students with elevated eating pathology. [file JCLP-82-1078-s001.docx]

**Supplemental Table 1.**

Relative weights analysis examining the contributions of gastrointestinal-specific hypervigilance and anxiety to purging in a sample of undergraduate students (n▒=▒309) with elevated weight and shape-oriented eating pathology.

|  | **Raw relative**  **Weight**  **Estimate [95% CI]** | **95% CI for significance of relative weight** |
| --- | --- | --- |
| Hypervigilance | .008 [.001, .021] | -.028, .018 |
| Anxiety | .032 [.006, .084] | -.006, .078 |
| R^2^ | .040 |  |
| Hypervigilance | .006 [.001, .010] | -.009, .018 |
| Anxiety | .019 [.006, .059] | .003, .070 |
| Visceral Sensitivity Index | .029 [.006, .070] | .004, .076 |
| R^2^ | .053 |  |

*Note.* The raw relative weight indicates the proportion of variance in purging that is explained by each predictor.

**Supplemental Table 2.**

Relative weights analysis examining the incremental validity of the unique modified EHAS Anxiety subscale items over and above the modified EHAS Hypervigilance subscale and the Visceral Sensitivity Index in explaining restricting (full sample, n▒=▒380) and purging (shape/weight-oriented eating pathology subsample, n▒=▒309).

|  | **Raw relative**  **Weight**  **Estimate [95% CI]** | **95% CI for significance of relative weight** |
| --- | --- | --- |
| **Restriction (n**▒=▒**380)** |  |  |
| Hypervigilance | .006 [.001, .013] | -.029, .014 |
| Anxiety – unique items | .013 [.003, .029] | -.019, .029 |
| Visceral Sensitivity Index | .039 [.010, .081] | .001, .080 |
| R^2^ | .058 |  |
| **Purging (square root transformed; n**▒=▒**309)** |  |  |
| Hypervigilance | .006 [.001, .010] | -.029, .013 |
| Anxiety – unique items | .019 [.001, .050] | -.001, .081 |
| Visceral Sensitivity Index | .036 [.009, .080] | -.009, .051 |
| R^2^ | .061 |  |

*Note.* The raw relative weight indicates the proportion of variance in purging that is explained by each predictor.

**Supplemental Table 3**.

Exploratory relative weights analysis examining the contributions of gastrointestinal-specific hypervigilance and anxiety to binge eating and cognitive restraint in a sample of undergraduate students (n▒=▒309) with elevated weight and shape-oriented eating pathology.

|  | **Raw relative**  **Weight**  **Estimate [95% CI]** | **95% CI for significance of relative weight** |
| --- | --- | --- |
| **Binge Eating** |  |  |
| Hypervigilance | .011 [.002, .029] | -.007, .036 |
| Anxiety | .041 [.010, .086] | .008, .092 |
| R^2^ | .052 |  |
| Hypervigilance | .008 [.002, .015] | -.016, .017 |
| Anxiety | .024 [.007, .049] | .0004, .053 |
| Visceral Sensitivity Index | .056 [.020, .103] | .017, .107 |
| R^2^ | .087 |  |
| **Cognitive Restraint** |  |  |
| Hypervigilance | .0001 [0, 0] | -.057, .003 |
| Anxiety | .0001 [0, 0] | -.057, .003 |
| R^2^ | <.001 |  |
| Hypervigilance | .0001 [0, 0] | -.044, .005 |
| Anxiety | .0001 [0, 0] | -.043, .005 |
| Visceral Sensitivity Index | .0001 [0, 0] | -.044, .005 |
| R^2^ | <.001 |  |

**Evaluating Differences in Upper and Lower Gastrointestinal Symptoms**

**Supplemental Table 4.**

Correlations between gastrointestinal symptoms and eating disorder symptoms in a sample of undergraduate students with elevated eating pathology.

|  | Restriction  N▒=▒380 | Purging (Square Root Transformed)  N▒=▒309 |
| --- | --- | --- |
| *Upper GI Symptoms* |  |  |
| Heartburn/regurgitation^a^ | .281** | .254** |
| Nausea/vomiting^a^ | .372** | .313** |
| Postprandial Fullness/early satiety^a^ | .555** | .187* |
| Upper abdominal pain^a^ | .221** | .261** |
| *Lower GI Symptoms* |  |  |
| Lower Abdominal Pain^a^ | .204** | .155* |
| Bloating/distension^a^ | .043 | .268** |
| Abdominal Symptoms^b^ | .228** | .306** |
| Rectal Symptoms^b^ | .157** | .217** |
| Stool Symptoms^b^ | .185** | .226** |

Note: **p*▒<▒.01; ***p*▒<▒.001

^a^ subscale came from the Patient Assessment of Gastrointestinal Symptom Severity Index (Rentz et al., 2004)

^b^ subscale came from the Patient Assessment of Constipation Symptom Severity Index (Frank et al., 1999)

Overall, the Visceral Sensitivity Index, which was developed in a lower GI sample, had the largest relative weights with eating disorder behaviors. This might indicate that lower gastrointestinal symptoms are more salient to eating pathology. To test this explanation, the strength of correlations between eating disorder behaviors and upper GI symptoms and eating disorder behaviors and lower GI symptoms were compared. Restriction was more strongly correlated with the upper GI symptom heartburn/regurgitation than the lower GI symptoms bloating/distension (z▒=▒4.01, *p* <.001) and rectal symptoms (z▒=▒2.12, *p*▒=▒.03). Restriction was more strongly correlated with the upper GI symptom nausea/vomiting than the lower GI symptoms lower abdominal pain (z▒=▒3.28, *p* <.001), bloating/distension (z▒=▒5.57, *p* <.001), abdominal symptoms (z▒=▒2.93, p <.01), rectal symptoms (z▒=▒3.79, *p* <.001), and stool symptoms (z▒=▒3.27, *p* <.001). Restriction was more strongly correlated with the upper GI symptom postprandial fullness/early satiety than the lower GI symptoms lower abdominal pain (z▒=▒7.52, *p* <.001), bloating/distension (z▒=▒10.78, *p* <.001), abdominal symptoms (z▒=▒7.32, *p* <.001), rectal symptoms (z▒=▒7.88, *p* <.001), and stool symptoms (z▒=▒7.75, *p* <.001). Restriction was more strongly correlated with the upper GI symptom upper abdominal pain than the lower GI symptom bloating/distension (z▒=▒3.38, *p*▒<▒.001). All other correlations between restriction and upper GI symptoms and restriction and lower GI symptoms were not significantly different from other one another (*p*’s▒≥▒.08).

Purging was more strongly associated with the lower GI symptom constipation-related abdominal symptoms than the upper GI symptom postprandial fullness/early satiety (z▒=▒2.12, *p*▒=▒.034). Purging was more strongly correlated with the upper GI symptom nausea/vomiting than the lower GI symptom lower abdominal pain (z▒=▒2.63, *p*▒=▒.009). Similarly, purging was more strongly correlated with the upper GI symptom of upper abdominal pain than the lower GI symptom of lower abdominal pain (z▒=▒2.158, *p*▒=▒.03). All other correlations between purging and upper GI symptoms and purging and lower GI symptoms were not significantly different from other one another (*p*’s▒≥▒.11).
